# MENDEL: Morphologist and Mathematician Founder of Genetics – To Begin a Celebration of the 2015 Sesquicentennial of Mendel's Presentation in 1865 of his *Versuche über Pflanzenhybriden*

**DOI:** 10.1002/mgg3.127

**Published:** 2015-01-08

**Authors:** John M Opitz, Diana W Bianchi

**Affiliations:** 1 Departments of Pediatrics (Medical Genetics), Human Genetics, Pathology, Obstetrics and Gynecology, University of Utah School of MedicineSalt Lake City, Utah; 2 Mother Infant Research Institute, Tufts Medical CenterBoston, Massachusetts

## Introduction

**Figure 1:**
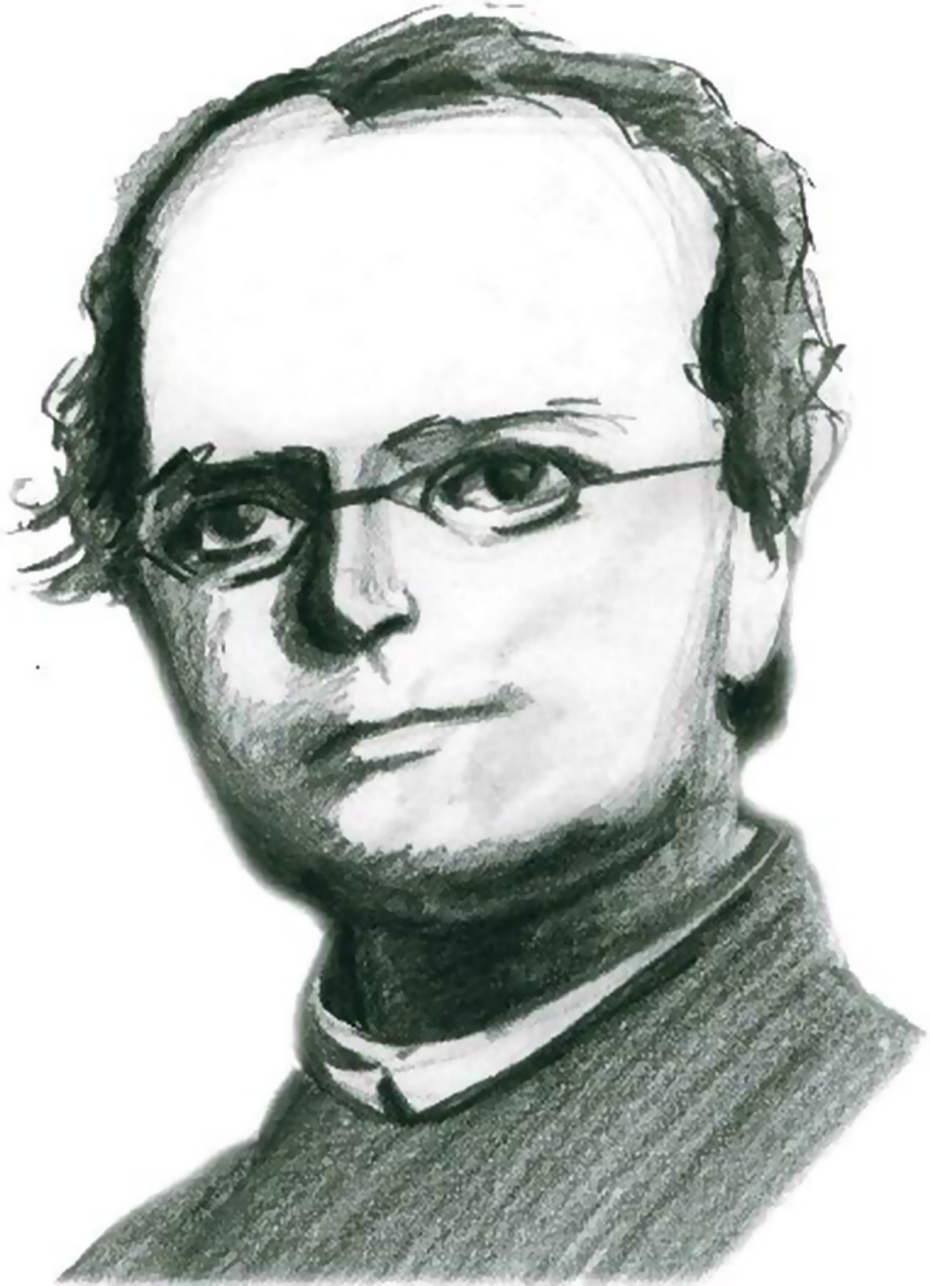
Pencil and ink sketch of Gregor Mendel (1822–1884) as imagined at the time he gave his lecture on February 8, 1865. Used with permission by the artist Claire Harper and provided by Dr. Sherri Bale, GeneDx.

**Figure 2:**
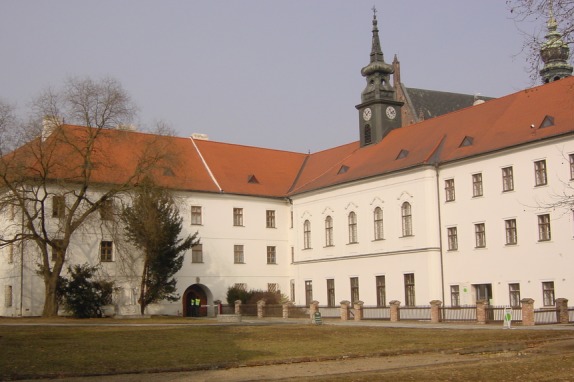
Partial view of the Königinkloster (“Queen's Cloister”), Abbey of St. Thomas of Altbrünn taken by Diana Bianchi in 2003. View from southwest. On the left, between two trees is the Charlemont monument to Mendel. The part of the building with clock tower houses the library and Mendel museum. The garden area between fence and building is the area where Mendel presumably had his experimental plot. Behind the clock tower is the gable of the church of St. Thomas from 1353.

Entering a school building, recently erected at state expenses, to attend a regularly scheduled evening session of a local and regional scientific association, few would give any thought to its foundation. Except perhaps those who watched the building go up in the downtown area of Brünn (Brno) in 1858/9 and who taught in it, as did Mendel (Fig.1). Thus, 150 years ago, on a cold clear (Iltis 1924) February night (2/8/1865) a small group of Augustinian monks, all priests, Mendel among them, made their way from their more or less permanent abode, the renowned, ancient abbey of St. Thomas, along the Johannesgasse to the new school building to hear Fr. Mendel present the results of his 8 years of research into plant hybrids, specifically crosses of several types of *Pisum sativum,* garden peas. The monastery with its beautiful Gothic church (Fig.2) was located in Altbrünn (Stare Brno), incorporated into Brünn, then as now the capital of Moravia. At the time, Moravia was in the Austrian Empire in that portion of Silesia left to Austria after the three wars between Frederic the Great and Empress Maria Theresia. Later it was in Czechoslovakia, and at present is in the Czech Republic.

Entering the *Oberrealschule*, the monks and their secular fellow members and guests of the Society of Natural Sciences (*Naturforschender Verein)* left their top hats, canes, and capes in the lobby, took their seats close to the stove, noting the officers of the Association moving forward to where Mendel had seated himself in the front row. The Vice President of the Association, the distinguished botanist Carl (or Karl) Theimer, announced the evening's agenda and asked the Association's equally accomplished secretary, the botanist and astronomer Gustav V. Niessl to introduce the speaker, Mendel, and invited him to give the first of his two presentations on Experiments in Plant Hybridization *(Versuche über Pflanzenhybriden)*. The second, Mendel's summary and conclusion, was given 1 month later (3/8/1865).

Thus, more or less imaginatively retold, occurred an event, surely equal in importance in the history of Western Biology to the presentation just 7 years earlier by Darwin's friends and colleagues to the Linnean Society of London (Slotten 2004) on the *theory* of descent with modification through the action of *natural selection* proposed by Wallace and Darwin.

## Mendel and Darwin

In contrast to Darwin, Mendel, quite aware of the theory of evolution, presented *facts*, not theory, *on inheritance*, discovered not through natural selection but by *artificial selection*, that is, by means of plant hybridization. When Darwin finally published in 1859 he left a substantial tome: “*On the Origin of Species by Means of Natural Selection, or the Preservation of Favored Races in the Struggle for Life*,” the argument (for natural selection) sustained by and meant to persuade principally by the sheer weight of a huge body of facts and data and inferences drawn from them. The issue it addressed, was condensed into a single word: “evolution” urged upon a reluctant Darwin by Herbert Spencer and used only once in 1859 (“evolved”), in the last sentence of The Origin. Darwin, a 19th century morphologist, was rather conscious of its use until recently in an embryological context. Darwin was equally nimble and expert in adducing animal *and* plant examples in his book; Mendel, also a 19th century morphologist, confined himself to plants in his presentation, although later he performed experimental breeding with bees. For Darwin, artificial selection was a means of hurrying up natural selection in producing the immense variety of domesticated animal and plant forms from one or more than one ancestor. Mendel dealt with both, wild forms collected around Brünn (i.e., the hawkweed, *Hieracium*, which later caused him difficulties in validating the conclusions of his earlier experiments) and forms under cultivation, mostly for practical agronomical (decorative and edible) purposes. Darwin postulated that it took millennia and hundreds of generations for natural selection to lead to a new species, while Mendel studied modification of plant forms from well-established parent stock in only a few generations. Mendel was fully aware of Darwin's work, as witnessed by his many annotations in the 1863 German translation of Darwin's works preserved to this day in the extensive monastery library and by the use of the word *Evolution* twice in his subsequently published (1866) paper that summarized both talks. Sadly, the converse is not true, despite the fact that Darwin's first cousin, Francis Galton, wrote to him in 1875 and 1876, asking him to grow peas to test Mendel's laws. Galton also recommended that Darwin read W. O. Focke's *Pflanzenmischlinge*, which was published in 1881.

The many words used by Darwin in the first edition of his seminal “On the Origin…” ignited as much opposition and confusion as any prior eureka in biology. Bateson, Mendel's staunchest and most verbal protagonist in England, used Mendelism to bludgeon natural selection acting on generationally accrued, infinitesimal changes, championing instead “sports”, mutations of major developmental effect (e.g., “homoeotic” ones, Bateson 1894) as evolution's raw material. It was not until 1918 that Fisher^1918^ reconciled Mendelian and Galtonian inheritance thereby initiating the genetic study of evolution making *genetics* and *descent* biologically compatible and complementary disciplines. That was almost 60 years after “The Origin…” Darwin lacked genetical and mathematical foundations to explain descent with modification; he was (in our opinion) too prolix, occasionally inconsistent, confused, and contradictory, postulating at last (again after Hippocrates) pangenesis and ending up a Lamarckian (someone whom he had damned roundly on previous occasion).

By contrast, Mendel was concise, distilling the essence of his evidence and argument into just 47 pages with far fewer words than Darwin required to make his point. When Mendel's efforts to have the respected botanist Karl Wilhelm von Nägeli at the University of Munich repeat his *Pisum* work failed (Correns 1905) and ended in utter frustration, he went on with life and work as the abbot of his community tentatively confident that “his time would [still] come.” When it came, 16 years after his death and 34 years after the publication of *Versuche,* it occasioned initial amazement, immediate acceptance and the kind of intellectual joy and pleasure attendant on and reserved for only those few truly great eurekas of Western Biology effective in reshaping our worldview, akin to the joy occasioned by a great, beautiful, unexpected gift. Not just something given and taken for granted as self-evident, but a stimulus so powerful in botany, zoology, and medicine as to occasion an intellectual revolution akin to and equal in effect to Darwinism (Iltis 1924). Iltis put it nicely: Mendelism is the atomic theory of life (translation). Thus, when Arnold Lang (1914) of Zürich made a summary of all of Mendelism in zoology up to the beginning of the First World War, he required no less than 892 pages for Part I; we do not know if Part II was ever published. In retrospect, it seems hardly believable that such a huge amount of biology involving humans, animals, and plants wanted to and needed to be subsumed under the head of Mendelism so rapidly after its startling birth and establishment as a biological discipline.

## Mendel: Morphologist and Mathematician

Mendel not only observed natural phenomena, as a well-trained mathematician and physicist, he applied mathematical theories to interpret his biological experiments and to plan new ones.

**Figure 3:**
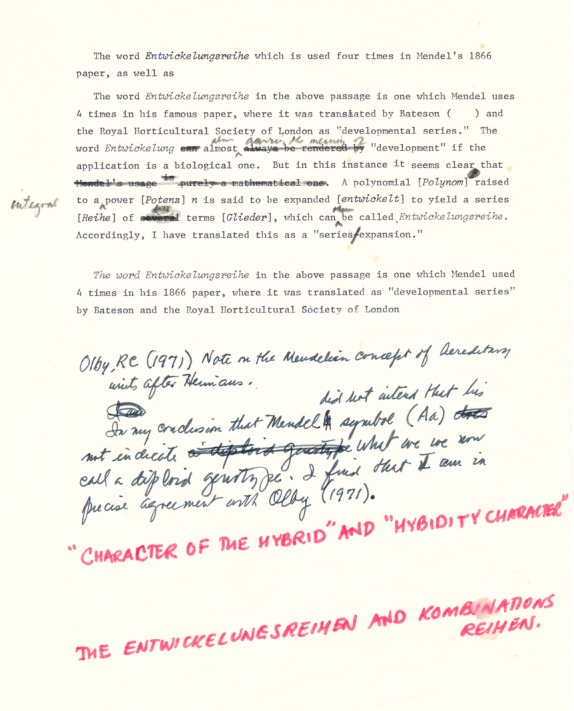
Note prepared by the late Charles W. Cotterman, then of the University of Wisconsin on the term *Entwicklungsreihe* used by Mendel. Date ? Opitz papers.

Iris Sandler (2000) was not far off when she emphasized the concept of *development* (*Entwicklung*) in Mendel's paper, except that she still confuses (as do Stern and Sherwood 1966) *Entwicklung*, the biological entity, with *Entwicklung* the mathematical entity. As the late C.W. Cotterman, then also at the University of Wisconsin, made clear at one time (Fig.3): “…in this instance it seems clear that… [A] polynomial (*Polynom*) raised to a power (*Potenz*) n is said to be expanded (*entwickelt*) to yield a series (*Reihe*) of terms (*Glieder*) which can then be called *Entwickelungsreihe* [sic] (i.e., (A + b)^2^ = A^2^ +2Ab + b^2^). Accordingly, I [Cotterman] have translated this as a ‘series expansion.’”^1^ And in working on a renewed translation of Mendel's *Versuche* from the original, our preference will be “polynomial expansion”, exactly. Iris Sandler's confusion is no different from that of William Bateson (1902) and the Royal Horticultural Society when they translated this as “developmental row,” *Entwicklungsreihe* having no meaning in German biology.

Near the beginning of the 19th century Goethe (and Burdach in 1800) introduced the term *Morphologie* into biology as science of the form, formation, transformation, and (after Meckel 1812) the malformation of living organisms (Opitz 2004). Form: anatomy, zootomy; formation: embryogenesis, embryology; transformation: evolution (after the Fr. t*ransformisme*). With respect to malformation (*Missbildung*): Meckel said (Opitz et al. 2006) they were “*not* contrary to nature,” and like their then normal anatomical counterparts, were quite limited in type and range since “nature is *not* infinitely variable,” being merely arrests of normal development or, what later came to be called atavisms (Darwin's reversions), abnormal in humans, normal in other species. This limitation is now referred to as “developmental constraint.”

This view of nature, whether philosophical or strictly factual, became so universally understood and entrenched in European thought in the 19th century as to make its explicit invocation as methodological approach to any and all work in biology redundant. Mendel worked on *form* (tall, short; round, wrinkled, etc.) and its *formation* in the hybrids, as practical an approach to botanical study and the exploration of plant development as that of his father, an expert fruit tree horticulturalist, and that of his mother, a professional gardener's daughter who loved ornamental flowers. Thus, Mendel did not have to mention morphology explicitly. His presentation made it abundantly clear that morphology was the epistemological basis of his botanical and later apicultural investigations. In part, this view of nature (Merz^1976^, 1904–1912, facsimile 1976) was acquired “osmotically” through an 8-year contact with his in-house Augustinian brother and friend, the historian and philosopher Fr. Franz Theodor Bratranek, a confidant of Goethe's daughter-in-law and her sons (Iltis 1924), with access to the Weimar Goethe archives and an editor of Goethe papers. Thus, it can be concluded that in the Altbrünn convent at mid-century Goethe and morphology permeated the intellectual atmosphere as completely as oxygen does the air. And Mendel's initial approach to a causal analysis of development in plants arose out of very real morphology, not out of his imagination (Di Trocchio 1991), even less out of a preconceived notion of plant genetics.

## Whence Mendel?

On 17 April 1850, when he was 28 years old, Mendel submitted a brief autobiographical note together with other documents to the examination commission for high school teachers in Vienna (Iltis 1924). It breathes Mendel's humility and honesty. Thereafter, Mendel did not indulge in autobiographical documentation. How one wishes that Mendel's two physician nephews had undertaken at least an oral history while their uncle was living and they were in frequent contact with him. Mendel made their education possible in loving gratitude to his younger sister who had gladly relinquished her dowry, or part of it, so that Mendel could complete his own (education). In 1902, when Mendel would have been 80, a memorial tablet was dedicated in Heinzendorf (Hynčice, Northern Moravia), Mendel's birth village, and affixed to the local fire station, where he had organized a fire brigade. The memorial address on that occasion was given by Dr. Alois Schindler, the older of Mendel's two nephews whom he supported during medical school. This necessarily condensed address (Schindler 1902) was printed privately and was a modest beginning of the Mendel historiography that continues to this day. This historical process was severely impaired at its beginning by Mendel's successor as abbot who destroyed virtually all of Mendel's papers after his death. On the whole though, most historians of Mendelism initially rely on Hugo Iltis of Brünn, whose biography of Mendel (1924) was thoroughly researched and beautifully written, including an exacting analysis of Mendelism to that date. Dr. Iltis tells charming stories of Mendel as animal lover, for all except snakes, who kept a fox tied up in the morning and released at night, and who made a startling acquaintance with his future pet hedgehog who had bedded down for the night in one of his boots.

The two monographs of Orel (1984, 1996) are indispensable for Mendel's studies; more recently Weiling (1991), published a summary of his decades-long studies of personal and professional aspects of Mendel's life.

When he became abbot, Mendel must have had occasional moments of utter astonishment at the contrast between his early life as a peasant's son, whose father still labored under the corvée and whose incapacitation while felling trees in the lord's forest put a severe crimp on the continuation of Mendel's education – and his present, relatively opulent life as prelate of a wealthy monastery. His attitude toward very hard work on his own and the lord's land never left Mendel, who did not allow himself a moment of rest after beginning monastic life, always giving the very best of himself as educator (physics, mathematics, natural history, German, Latin, and Greek), as practical horticulturist, investigator of plant morphology, meteorologist, astronomer, member of the Moravian legislature, administrator of the wealthiest and most renowned monastery of Moravia and of the mortgage bank of Moravia, member of some two dozen learned societies and associations, patron of the arts, and benefactor of the poor.

## Mendel Falsified?

It would be naïve to think that the data in his *Versuche* were the only ones available to Mendel for his presentation or manuscript in 1865/6. Mendel was an extremely hard worker and it can be assumed without risk of contradiction that he performed more, perhaps many more experiments than necessary to make his point. As an outstanding teacher, renowned for his didactic skills, he evidently used great care in sorting his material to make as convincing a case as possible. Nowhere in the records from over a half century have we ever come across even a faint hint questioning Mendel's probity or honesty. This is not to be assumed just because he was a priest, but it was known and universally acknowledged by his contemporaries that Mendel was the very essence of integrity, meriting, after all, presidency of a bank.

Also, we must not make the mistake of applying present day standards of data analysis and presentation to the style and standards of the 1860s; Mendel did not write for the editors of *Nature*. The difference is of the essence and it is cultural, not a failure to correctly apply Chi-squared tests, which after all were not developed until after Mendel's death. Mendel's data were presented not to deceive, but to clarify and to teach. Furthermore, his writings, especially to von Nägeli as early as 1866, suggest that he was asking for others to validate his experimental work.

Fisher (1936) concluded: “There can, I believe, now be no doubt whatever that his report is to be taken entirely literally, and that his experiments were carried out in just the way and much in the order that they are recounted.” But: “…the data of most, if not all, of the experiments have been falsified so as to agree closely with Mendel's expectations.”

Sewall Wright's (1966) reaction: “The most serious evidence for fraud by Mendel, presented by Fisher, is the very close agreement to a ratio of….” Wright redid (some of) Fisher's calculations, agrees with his results, but cautions: “I do not think that Fisher allows enough for the cumulative effect on *Χ*^2^ of a slight subconscious tendency to favor the expected result in making tallies. Mendel was the first to count segregants at all. It is rather too much to expect that he would be aware of the precautions now known to be necessary for completely objective data. Anyone who doubts the difficulty in making repeatable counts should read chapter 5 in Pearl's *Introduction to Medical Biometry*. He reports an experiment in which 15 trained observers obtained extraordinary differences in sorting and counting the same 532 kernels of corn. Checking of counts that one does not like, but not of others, can lead to systematic bias toward agreement. I doubt whether there are many geneticists even now whose data, if extensive, would stand up wholly satisfactorily under the *Χ*^2^ test. “Mendel's perplexing ratios of round × angular and yellow × green… would hardly have been reported by one bent on fraud”. Wright concludes: “Taking everything into account, I am confident, however, that there was no deliberate effort at falsification.”

Similarly, Sturtevant (1985): “Perhaps the best answer – with which Fisher would have agreed – is that after all, Mendel was right.”

Weiling (1991, 1993/1994) has examined this issue on several occasions concluding that “…since it was determined that the statistical model underlying the Chi square test is inappropriate for the conditions underlying Mendel's segregation data, … Fisher's conclusion does not apply.” Edwards (1993): “As to the controversy over his goodness-of-fit findings …the less said the better. The only people on whom it reflects badly are those writers who have viewed it as casting doubt on the scientific integrity of either Mendel or Fisher.”

## Conclusions

**Figure 4:**
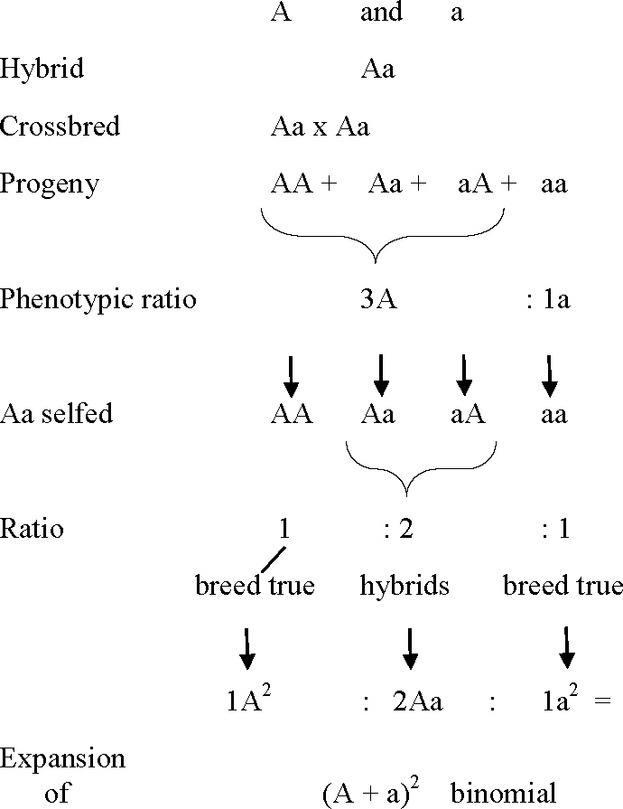
Succinct summary of Mendelism to Mendel's death in 1884.

Assuming the sexuality of plants, the equivalence of pollen and ovule in the production of offspring and the self-evident transmission of plant traits over generations (“inheritance”), the following conclusions may be drawn from elementary Mendelism involving pairs of contrasting traits (Fig.4).

Exponent: Number of traits; **a** having the property of becoming latent (“recessive”) in the hybrid dominated by the “domineering” **A** thus designated subsequently the dominant (partner) trait.

The following 10 inferences may be drawn from these results:

1. Determinants of **A **and* ***a** are concrete material, particulate entities…
2. .…which are apparently assembled in (a pairwise manner) in hybrids without merger or loss of material integrity…
3. .…as shown by their intact individual reappearance (“segregation”, Bateson and Saunders 1902) in the germ cells and
4. .…the reappearance of the recessive trait in the progeny of the hybrids.
5. The determinants of **A** and **a** must be equivalent in structure and function to judge from their 1: 1 occurrence in the offspring of the hybrids…
6. .…regardless whether carried by pollen or ovules.
7. Determinants of **A** and **a** are the morphological units of function affecting the development of the character traits; as such…
8. .…they may be pleiotropic (Plate 1910) and …
9. .…have to be taken into account of any trait affecting evolution since…
10. .…they may be responsible, wholly or in part, for those traits deemed “desirable” by the breeder, thus leading in the short term to the appearance of “new” races, breeds and cultivars by artificial selection.

Fisher (1936), referring to Focke's *Pflanzenmischlinge* which, like Nägeli, completely missed the epochal implications of Mendel's work, stated “…the learned author (Focke) having overlooked, in his chosen field, experimental researches *conclusive in their results, faultlessly lucid in presentation, and vital to the understanding not of one problem of current interest, but of many*” (italics added)”. In retrospect, no one can disagree with this assessment of Mendel's work.

From the surviving records, the testimony of contemporaries and the evidence collected by historians closest to Mendel in time (but all after 1900) it seems safe to conclude that above all Mendel was a “good” man in the best meaning of the word. Not just by ecclesiastical standards, Mendel being only one of many abbots in Western Catholicism, but first of all by the astonishing outpouring of grief at his death, for example, by his former students, fellow pedagogues and by the poor citizens of the town.

And what a legacy for an abbot (#) to bequeath to humanity, no hymns, no theological treatises, no sermons, no record of particular sanctity (indeed the opposite in his obstinate refusal to pay the church tax). Rather, a brief 47-page monograph on the progeny of plant hybrids, work conceived and executed over 10 years apparently without preconceived notions, and confined strictly to what we now call phenotypes and the behavior of their *formbildenden Elemente* in the germ cells. This astonishing epistemological restraint made it so easy for his successors, for example, De Vries, Correns, Tschermak, Bateson…to take the next step to “genotype”.

We agree with Olby (1966, 1979,1991) that Mendel did not set out to discover Mendelism, or to plan his laborious experiments accordingly, or to “falsify” his results so as to best fit expectations (Fisher 1936). We differ only slightly from Fisher in that Mendel probably did not stop counting when he had made his point, more or less exactly, but rather *selected* the best results from many. During the dreadful time of war after his presentations, the occupation of Brünn by some 50,000 Prussians in early 1866, and the ensuing cholera epidemic and food shortages Mendel had some time to reflect on his manuscript before its publication in 1866. He summarized his results in the didactically clearest manner, not to deceive but to impress as parsimoniously as possible. Additionally, his use of comparative *mathematics* to interpret *biology* was ahead of its time, having important parallels today in the use of computational biology to analyze big datasets. Mendel was a geneticist and genomicist *malgré lui*. In the 150 years since Mendel's oral presentations, we have taken the inferences drawn from his work so far as to begin an inventory of the genes in LUCA, an organism that lived some 3.6 billion years ago. LUCA became the ancestor of all living (and extinct) pro- and eukaryotes (Goldman et al. 2013; on LUCApedia). These astonishing advances and capabilities notwithstanding, Mendel would probably be content to have made a beginning at the phenome, or rather at phenomics, genomics having become one of those unanticipated consequences arising from the work of an astute man far ahead of his times.

## Footnotes

1 Cotterman, a colleague and friend of John M. Opitz in Madison for 18 years before his death, was not only an expert germanist but also mathematician, the equal of Mendel and for a while, the world pioneer and greatest authority on combinatorics and path coefficients applied to humans – specifically immunogenetics.
